# Comparison of conventional fluid management with PVI-based goal-directed fluid management in elective colorectal surgery

**DOI:** 10.1007/s10877-018-0163-y

**Published:** 2018-06-14

**Authors:** Sevim Cesur, Tülay Çardaközü, Alparslan Kuş, Neşe Türkyılmaz, Ömer Yavuz

**Affiliations:** 1 Department of Anesthesiology and Reanimation, Kocaeli University of Medical Faculty, Izmit, Kocaeli Turkey; 2 Department of General Surgery, Kocaeli University of Medical Faculty, Izmit, Kocaeli Turkey

**Keywords:** Pleth variability index, Goal directed fluid management, Colorectal surgery, Crystalloid fluid

## Abstract

Intraoperative fluid management is quite important in terms of postoperative organ perfusion and complications. Different fluid management protocols are in use for this purpose. Our primary goal was to compare the effects of conventional fluid management (CFM) with the Pleth Variability Index (PVI) guided goal-directed fluid management (GDFM) protocols on the amount of crystalloids administered, blood lactate, and serum creatinine levels during the intraoperative period. The length of hospital stay was our secondary goal. Seventy ASA I–II elective colorectal surgery patients were randomly assigned to CFM or GDFM for fluid management. The hemodynamic data and the data obtained from ABG were recorded at the end of induction and during the follow-up period at 1 h intervals. In the preoperative period and at 24 h postoperatively, blood samples were taken for the measurement of hemoglobin, Na, K, Cl, serum creatinine, albumin and blood lactate. In the first 24 h after surgery, oliguria and the time of first bowel movement were recorded. Length of hospital stay was also recorded. Intraoperative crystalloid administration and urine output were statistically significantly higher in CFM group (p < 0.001, p: 0.018). The end-surgery fluid balance was significantly lower in Group GDFM. Preoperative and postoperative Na, K, Cl, serum albumin, serum creatinine, lactate and hemoglobin values were similar between the groups. The time to passage of stool was significantly short in Group-GDFM compared to Group-CFM (p = 0.016). The length of hospital stay was found to be similar in both group. PVI-guided GDFM might be an alternative to CFM in ASA I–II patients undergoing elective colorectal surgery. However, further studies need to be carried out to search the efficiency and safety of PVI.

## Introduction

Intraoperative fluid management is quite important in terms of postoperative organ perfusion and complications [1]. Various complications such as acute renal failure, hypotension, arrhythmia, anastomosis leak may occur secondary to intraoperative hypovolemia whereas hypervolemia may cause pulmonary edema, postoperative pneumonia, prolonged mechanical ventilation, delayed wound healing, edema in the gastrointestinal system (GIS), and decreased GIS motility [2–5]. In the perioperative period, fluid therapy and gastrointestinal function may complement each other or complicate it. If fluid therapy is not optimal, it may cause delayed gastrointestinal function and avoid early oral intake. If gastrointestinal dysfunction develops in the perioperative period, it may lead to fluid and electrolyte loss and metabolic problems [6]. Thus, the intraoperative fluid management of the patient is very important. Different intraoperative fluid management protocols are in use for this purpose. Of these protocols, the most common one is conventional fluid management (CFM). Fluid replacement is managed according to clinical assessment and heart rate (HR), arterial blood pressure (ABP) and central venous pressure (CVP) monitorization. However, clinical studies indicate that changes in ABP cannot be used for the monitorization of stroke volume (SV) and cardiac output (CO) and that measuring CVP is not enough for estimating the fluid response [7, 8]. Another method is the goal-directed fluid management (GDFM) and it is based on individualized fluid management using the static (HR, CVP etc.) and dynamic parameters, [stroke volume variability (SVV), pulse pressure variability (PPV) etc.] [2, 9].

In the present study, we compared the CFM and Pleth Variability Index (PVI), a non-invasive parameter-based GDFM, in terms of intraoperative fluid management in patients undergoing elective colorectal surgery. Our primary goal was to compare the effects of both fluid management protocols on the amount of crystalloids administered, blood lactate, and serum creatinine levels during the intraoperative period. The length of hospital stay was our secondary goal.

## Materials and methods

This prospective, randomized study was conducted after obtaining the Kocaeli University Faculty of Medicine Ethics Committee approval (KU CREC 2015/99). Our study was registered with NCT03339895 on clinicaltrials.gov. Informed consent was obtained from all individual participants included in the study. Seventy ASA (American Society of Anesthesiology) I–II patients over the age of 18 years, who would undergo elective open colorectal tumor surgery were included. The exclusion criteria were determined as having a serious cardiac arrhythmia and peripheral artery disease, an ejection fraction below 30%, a pulmonary pathology preventing inhalation with a volume more than 6 ml/kg via mechanical ventilation and the presence of liver and renal dysfunction.

The patients were randomized by double-blind closed envelope method and divided into two groups: 35 patients were in the conventional fluid management group (Group-CFM) and 35 patients were in the PVI-based goal-directed fluid management group (Group-GDFM). When the patient was admitted to General Surgery Clinic mechanical bowel cleansing (45 ml solution consisting of sodium dihydrogen phosphate + disodium hydrogen phosphate content, applied orally twice each) was performed 1 day before the operation and a liquid regimen of 0.45% NaCl and 5% dextrose was administered at 35 ml/kg/24 h. It was allowed to receive clear fluid up to 2 h before anesthesia induction and up to 6 h solid uptake. The premedication was achieved with 0.03 mg/kg intravenous (i.v.) midazolam (Zolamid®, Defarma) and 0.9% NaCl infusion was initiated to all patients. The patients taken to the operating room were monitored for electrocardiography (ECG) in the standard DII derivation, HR, non-invasive blood pressure (NIBP) and peripheral oxygen saturation (SpO_2_). The induction of general anesthesia was achieved with fentanyl (Talinat®, Vem Pharma) 1 ${\upmu }$g/kg, thiopental (Pental® sodium, I.E. Ulagay) 5–7 mg/kg and rocuronium bromide (Myocron, Vem Pharma) 0.6 mg/kg. The patients were intubated with a cuffed endotracheal tube (ETT) (women 7.5 no ETT–men with 8.0 no ETT). Following the endotracheal intubation, all patients were ventilated with Drager Primus® (Draeger Medical AG & Co., Germany) anesthesia machine with the inspiratory/expiratory ratio of 1/2 in the volume-controlled mode of 8 ml/kg. The respiratory rate was initiated as 10 breaths/min in two groups and then it was set to achieve an end-tidal carbon dioxide (EtCO_2_) value between 35 and 40 mmHg. The end-expiratory positive pressure was not administered to the patients. The patients were all positioned in the supine or Lloyd-Davies position (Trendelenburg with legs apart) with an arm board for the test arm. Radial artery catheterization was performed in the non-dominant hand of all patients using a 20 G catheter for invasive blood pressure monitorization and arterial blood gas (ABG) analysis after induction. The anesthesia of the patients was maintained with a total of 3 l/min fresh gas flow in 40% O_2_–60% N_2_O mixture and with 1.0 MAC Sevoflurane (Sevorane®, Abbot) inhalation. When muscle relaxation and analgesia were required, i.v. boluses of rocuronium and fentanyl were administered.

In Group-CFM, a 7Fr. CVP measurement catheter (Arrow®_,_ International, USA) with three lumens 20 cm in length was inserted into the right jugular vein of the patients in an ultrasound-guided approach. The infusion was continued with 0.9% NaCl solution at the dose of 4–8 ml/kg/h after the induction of anesthesia. The intraoperative fluid infusion was performed by the same anesthesiologist according to the routine practice in our clinic by taking the parameters such as HR, mean arterial pressure (MAP), CVP and urine output into consideration. Hypotension was defined as a condition in which the MAP was below 65 mmHg or 30% below the baseline MAP of the patient. In this case, the speed of crystalloid infusion was increased, colloid (Gelofusine® Melsungen, Germany) infusion was initiated and in case of hypotension persistence, 5 $\text{mg}$ i.v. ephedrine was administered. Ephedrine was repeated every 5 min till the MAP was increased over 65 mmHg.

In group-GDFM, a pulse oximetry probe (LNOP® Adt; Masimo Corp., USA) was connected to the fourth finger of the hand in which there was not an arterial catheter in all patients and it was wrapped so that it would not be affected by the external light. The pulse oximeter was connected to the Masimo Radical 7 monitor (Masimo SET; Masimo Corp., USA) including the PVI software (version 7.0.3.3). PVI is automatically and continuously calculates the respiratory variations in the photoplethysmogram from data collected noninvasively via a pulse oximetry sensor. PI reflects the amplitude of the pulse oximeter waveform and is calculated as the pulsatile infrared signal (AC or variable component), indexed against the non-pulsatile infrared signal (DC or constant component).

$${\text{PI}}\,\left( \% \right)=\left( {{\text{AC}}/{\text{DC}}} \right) \times 100$$

$${\text{Using PI}},{\text{ PVI is calculated}}.{\text{ PVI}}=\left( {{\text{P}}{{\text{I}}_{{\text{MAX}}}}-{\text{ P}}{{\text{I}}_{{\text{MIN}}}}{\text{/P}}{{\text{I}}_{{\text{MAX}}}}} \right) \times 100\%$$

**Fig. 1:**
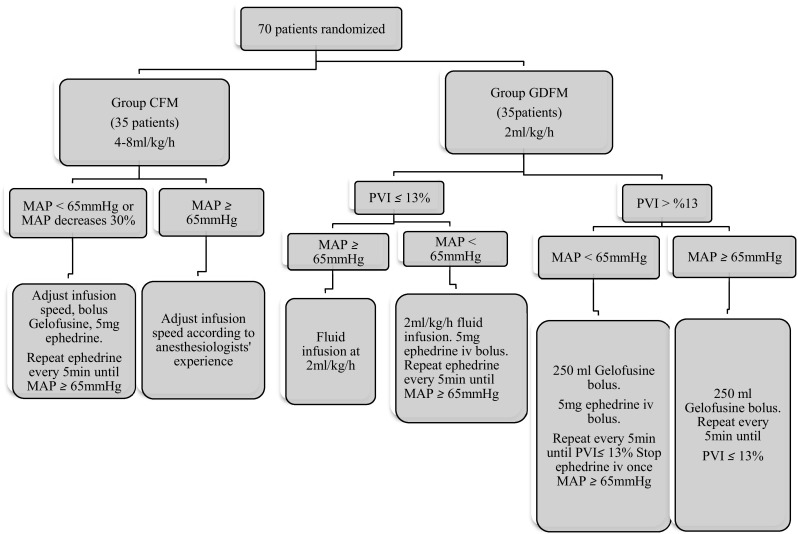
Study flow chart. *CFM* conventional fluid management, *GDFM* goal directed fluid management, *PVI* pleth variability index, *MAP* mean artery pressure, *iv* intravenous injection

It was awaited until the monitor values were stabilized and then PVI values were recorded. After the induction of anesthesia, 0.9% NaCl infusion at the dose of 2 ml/kg/h was resumed in the patients of Group-GDFM. If the PVI was higher than 13% for more than 5 min, a 250-ml bolus of Gelofusine® was administrated. If the PVI was still higher than 13% after the bolus infusion of fluid, it was repeated every 5 min until the PVI was less than 13%. Intravenous bolus of 5 mg ephedrine was given as needed to keep the mean arterial BP over 65 mmHg during this process. In the cases where PVI was less than < 13% and MAP < 65 mmHg, 5 mg iv ephedrine was applied and repeated every 5 min to keep MAP over 65 mmHg (Fig. 1).

The hemodynamic data and the data obtained from ABG [Ph, arterial oxygen pressure (PaO_2_), arterial carbon dioxide pressure (PaCO_2_), HCO_3_ level, hemoglobin level, blood lactate level] were recorded at the end of induction (0 h) and during the follow-up period at 1 h intervals. In the preoperative period and at 24 h postoperatively, blood samples were taken for the measurement of hemoglobin, Na, K, Cl, serum creatinine, albumin and blood lactate. Postoperative fluid management was performed according to the fluid protocol of the surgical clinic. In the first 24 h postoperatively, oliguria (urine output < 0.5 ml/kg), blood and blood product requirement, postoperative time to passage of stool (by defining with the number of day after the operation) and length of hospital stay were recorded.

### Statistical analysis

Ninety-seven, ASA I–II patients who underwent elective colorectal surgery between 2014 and 2015 were investigated retrospectively, which showed the amount of intraoperative crystalloid administered, with CFM was found as 2335 ± 906 ration. Power analysis estimated that 30 patients were needed for each group with the prediction that PVI-based GDFM would reduce the amount of crystalloid by 20%. However, considering the possibility of the patients to be excluded, we determined the number of patients to be 35 in each group. The statistical assessment was carried out with IBM SPSS 20.0 (SPSS Inc., Chicago, IL, USA) program package. The Kolmogorov–Smirnov Test was used for checking normal distribution. The numerical variables with normal distribution were expressed as mean ± standard deviation (SD) while the numerical variables without normal distribution were expressed as median (25th–75th percentile), and categorical variables were given as frequency (percent). The difference among the groups was determined by the student t-test for the numerical variables with normal distribution whereas it was determined with the Mann Whitney U Test for the numerical variables without normal distribution. For intra-group comparisons, the paired t-test and repeated measures ANOVA were used when the assumption of normal distribution was met while the Wilcoxon Signed Rank Test and Friedman two-way ANOVA were used when this assumption was not met. The associations among the categorical variables were assessed with the Chi square analysis. When p was < 0.05, it is considered sufficient for statistical significance.

## Results

The demographic and preoperative characteristics were similar in both groups (Table 1). The durations of anesthesia and surgery were similar as well. The amounts of intraoperative bleeding and administered colloid were similar. Intraoperative crystalloid administration and urine output were statistically significantly higher in CFM group (p < 0.001, p: 0.018). The end-surgery fluid balance was significantly lower in Group GDFM. In the intraoperative period, ephedrine was used in similar numbers of patients in both groups (Table 2). The number of patients including the third and fourth hours was not sufficient for statistical assessment, the intraoperative hemodynamic data and the data obtained from ABG were evaluated through the data in the first 2 h (Tables 3, 4). The HR was similar in both groups at the end of the first hours while it was significantly higher at 2nd hours in Group-GDFM (p: 0.026). In Group-CFM, both MAP and HR showed a significant difference compared to their post-induction levels at the end of second hour (p < 0.001; p < 0.001) (Table 3). Except for the lactate value at the 1st hour (p: 0.04), the values of Ph, PaO_2_, PaCO_2_, HCO_3_ and hemoglobin were similar between the groups (Table 4). Preoperative and postoperative Na, K, Cl, serum albumin, serum creatinine, lactate and hemoglobin values were similar between the groups (Table 5). The time to passage of stool was significantly short in Group-GDFM compared to Group-CFM (p = 0.016). The length of hospital stay was found to be similar in both groups (Table 6).

**Table 1:** Demographic and clinical characteristic of patients

	Group-CFM (n = 35)	Group-GDFM (n = 35)	P
Age (years)	62.31 ± 10.52	58.68 ± 14.41	0.233
Sex (female/male)	14/21	15/20	1.000
Weight (kg)	78.00 (71.00–86.00)	75.00 (61.00–80.00)	0.102
Height (cm)	173.00 (160.00–180.00)	170.00 (160.00–180.00)	0.557
BMI (kg/cm^2^)	26.86 ± 4.17	25.55 ± 4.96	0.237
ASA I/II	19/16	22/13	0.249

Data are presented as mean ± SD, median (25–75 percentile) or number of patients P < 0.05 for all the data

**Table 2:** Features of intraoperative surgery and anesthesia

	Group-CFM	Group-GDFM	p
Duration of anesthesia (min)	155 (135–200)	165 (135–195)	0.732
Duration of surgery (min)	140 (120–180)	150 (120–180)	0.876
Blood loss (ml)	250 (100–400)	200 (100–400)	0.662
Intraoperative crystalloid (ml)	1946 (1500–2500)	900 (800–1060)	< 0.001*
Intraoperative colloid (ml)	0 (0–500)	250 (0–500)	0.405
Intraoperative urine output (ml)	400 (250–600)	300 (200–400)	0.018*
Fluid balance (ml)	1400 (960–2250)	620 (410–1000)	< 0.001*
Amount of intraoperative ephedrine (mg)	7.50 ± 2.57 (n*=18)	5.00 ± 6.20 (n*=12)	0.672

Data are presented as median (25–75 percentile)

n* = Number of patients received ephedrine treatment

*P < 0.05, (Mann–Whitney U test)

**Table 3:** Intraoperative hemodynamic data

	Group-CFM	Group-GDFM	p
MAP (mmHg)			
0 h	98.57 ± 17.74	91.11 ± 13.24	0.050
1 h	81.54 ± 12.72	85.02 ± 13.72	0.275
2 h	86.00 (80.00–96.00)	82.50 (75.00–89.00)	0.524
p	< 0.001**	0.207	
HR (/min)			
0 h	80.00 (72.00–99.00)	84.00 (78.00–90.00)	0.814
1 h	79.91 ± 18.15	79.71 ± 14.44	0.959
2 h	73.27 ± 16.49	82.43 ± 15.50	0.026*
p	< 0.001**	0.139	

Data are presented as mean ± SD or median (25–75 percentile) P < 0.05 for all the data (Mann–Whitney U test, Repeated Measures ANOVA test, Wilcoxon Signed Rank Test and Friedman two-way ANOVA)

*MAP* mean artery pressure, *HR* heart rate, *0 h* the end of induction, *1 h* induction after 1 h, *2 h* induction after 2 h

*Statistical significance in cross-group comparisons

**Statistical significance when compared to 0 h values at the end of 2nd hour

**Table 4:** Intraoperative blood gas data

	Group-CFM	Group-GDFM	p
pH			
0 h	7.43 ± 0.04	7.42 ± 0.04	0.562
1 h	7.38 ± 0.05	7.38 ± 0.04	1.000
2 h	7.36 ± 0.03	7.36 ± 0.04	0.681
p	< 0.001**	< 0.001**	
PaO_2_ (mmHg)			
0 h	177.28 ± 70.80	171.32 ± 58.55	0.703
1 h	116.02 ± 32.92	126.30 ± 32.25	0.191
2 h	123.00 (107.00–147.50)	129.00 (110.50–161.00)	0.383
p	< 0.001**	0.001**	
PaCO_2_ (mmHg)			
0 h	33.99 ± 3.30	32.42 ± 3.33	0.053
1 h	34.97 ± 3.68	34.08 ± 3.36	0.562
2 h	34.50 (32.80–37.15)	35.90 (32.75–38.07)	0.405
p	0.786	0.011**	
Bicarbonate (mmol/l)			
0 h	23.96 ± 2.23	23.00 ± 2.34	0.084
1 h	22.05 ± 2.13	21.50 ± 2.19	0.287
2 h	20.83 ± 2.04	20.76 ± 2.28	0.891
p	< 0.001**	< 0.001**	
Hemoglobin (g/dl)			
0 h	12.00 (10.70–13.20)	11.40 (10.40–12.10)	0.211
1 h	11.90 (10.30–13.40)	11.50 (10.60–13.10)	0.892
2 h	11.88 ± 1.47	11.87 ± 1.78	0.981
p	0.200	0.359	
Lactate (mmol/l)			
0 h	0.70 (0.60–0.90)	0.70 (0.60–1.00)	0.270
1 h	0.80 (0.70–0.90)	0.90 (0.70–1.10)	0.040*
2 h	0.90 (0.70–1.00)	1.00 (0.80–1.20)	0.061
p	0.002**	< 0.001**	

Data are presented as mean ± SD or median (25–75 percentile) P < 0.05 for all the data (Mann–Whitney U test, Repeated Measures ANOVA test and Wilcoxon Signed Rank Test and Friedman two-way ANOVA)

*0 h* the end of induction, *1 h* induction after 1 h, *2 h* induction after 2 h

*Statistical significance in cross-group comparisons

**Statistical significance when compared to 0 h values at the end of 2nd hour

**Table 5:** Physiologic status on admission to preoperative and postoperative

	Group-CFM	Group-GDFM	p
Na_(mEq/l)preop_	140.00 (139.00–141.00)	140.00 (138.00–142.00)	0.986
Na_postop_	139.00 (138.00–143.00)	140.00 (138.00–143.00)	0.148
p	0.778	0.151	
K_(mEq/l)preop_	4.38 (4.05–4.68)	4.26 (3.73–4.55)	0.229
K_postop_	3.54 (3.22–3.73)	3.50 (3.20–3.77)	0.764
p	< 0.001**	< 0.001**	
Cl_(mEq/l)preop_	105.37 ± 3.69	105.37 ± 4.93	0.996
Cl_postop_	108.00 (104.00–111.00)	107.00 (104.99–112.00)	0.877
p	0.001**	0.001**	
Albumin_(g/dl)preop_	3.94 ± 0.47	4.06 ± 0.54	0.338
Albumin_postop_	3.19 ± 0.51	3.24 ± 0.54	0.688
p	< 0.001**	< 0.001**	
Creatinine_(mg/dl)preop_	0.79 (0.65–0.94)	0.79 (0.67–0.82)	0.729
Creatinine_postop_	0.76 (0.63–0.99)	0.75 (0.63–0.94)	0.991
p	0.808	0.501	
Lactate_(mmol/l)preop_	0.70 (0.60–0.90)	0.70 (0.60–1.00)	0.270
Lactate_postop_	1.70 (1.30–2.33)	1.55 (1.22–1.99)	0.445
p	< 0.001**	< 0.001**	
Hemoglobin_(g/dl)preop_	12.00 (10.70–13.20)	11.40 (10.40–12.10)	0.211
Hemoglobin_postop_	12.02 ± 1.48	11.48 ± 1.45	0.128
p	0.725	0.577	

Data are presented as mean ± SD or median (25–75 percentile) P < 0.05 for all the data (t test; Wilcoxon Signed Rank Test and Friedman two-way ANOVA)

*Na* sodium, *K* potassium, *Cl* chlorine, *preop* preoperative, *postop* postoperative

**Statistical significance when compared to preoperative and postoperative values

**Table 6:** Postoperative hospitalization, recovery of gut function

	Grup-CFM	Grup-GDFM	p
Bowels recovery (days)	5.00 (5.00–6.00)	4.50 (3.00–6.00)	0.016
Postoperative hospitalization (days)	6.00 (6.00–7.00)	6.00 (5.00–7.00)	0.331

Data are presented as mean ± SD or median (25–75 percentile) P < 0.05 for all the data (Mann–Whitney U test)

## Limitation of the study

We determined the primary goal of this study as the amount of intraoperative fluid volume and established 35 patients were needed for each group; if postoperative complications, the length of hospital stay determined as the primary goal, perhaps our numbers of the each groups could be different. Another limitation, we did not follow the effect of PVI guided fluid management on the patient’s long-term prognosis and correlation with blood lactate level, and the sample size was small. The duration of postoperative follow-up of the patients could be longer and the postoperative complications could be examined in more detail. Another limitation of the study was that the investigator following the intraoperative period was not blind.

## Discussion

According to the results of our study, when the PVI-based GDFM was compared with CFM in patients undergoing elective colorectal surgery, the former reduced the intraoperative total crystalloid administration and shortened time to stool passage, but did not show any effect on the length of hospital stay.

Although comprehensive studies have been conducted on fluid management in the intraoperative period, the “correct fluid volume” remains still unclear. The only scientific evidence available is that excessive fluid load seems like a wrong strategy [10]. The fluid management depends on the preoperative volume status, comorbidity factors, age of the patient, anesthesia technique, and type of surgery. Restrictive fluid management (RFM) and GDFM are recommended for high-risk patients undergoing high- and moderate-risk surgeries while liberal fluid management (LFM) may be preferred for low-risk patients undergoing low- and medium-risk surgeries [1, 11, 12].

The results of the studies on fluid management in colorectal surgery show variabilities [13]. The first reason for this is that primary goals differ among the studies. For example; fluid management is addressed in terms of postoperative complications, RFM reduced complications. On the other tissue perfusion was targeted, LFM increased tissue perfusion [14, 15]. Holte et al. [16] stated that RFM preserved postoperative pulmonary functions better compared to LFM while Nisanevich et al. [5] reported that RFM reduced postoperative complications but did not affect mortality.

The second reason for the different results in the studies is the differences in the classification and definition of intraoperative fluid management. The fluid management regimes were classified as CFM, RFM and GDFM in one source whereas in another source, it was classified as LFM, RFM and GDFM [10, 17]. In fact, there is no standardized fluid volume even for LFM and RFM. For example, Abraham-Nordling et al. [18] defined LFM with the crystalloid at 7 ml/kg/h while Holte et al. [16] defined the co-administration of crystalloid at 7 ml/kg/h and colloid at 7 ml/kg/h as RFM. The primary goal in the CFM replacement of losses, during fasting period in the intraoperative period. However, in patients without cardiac risk, the patients were determined to be normovolemic after 10 h of fasting [19]. The presence of the third space, one of the key components of CFM, is still being discussed [20]. Therefore, CFM can cause hypervolemia [4, 21–23]. In elective colorectal surgery, perioperative excessive fluid was reported to cause pneumonia and respiratory failure, increase the work of renal diuresis, intestinal edema, inhibit bowel movements, postoperative ileus, reduction in tissue oxygenation and delayed wound healing due to increased cutaneous edema [4]. The studies on GDFM revealed results generally in favor of GDFM in terms of respiratory risks, renal and gastrointestinal complications, restoration time of bowel function, and discharge time from the hospital [17, 24–26]. In the meta-analysis by Pearse et al. [27], GDFM and CFM were compared and the incidence of complications such as postoperative infections and 30-day mortality was found to decrease with GDFM while the length of hospital stay was relatively increased. Although the bowel functions were restored in a shorter period of time with GDFM in our study, we could not find any stastical relation between restored bowel function duration after surgery and length of hospital stay.

Blood lactate levels provide an indirect but sensitive measure of organ perfusion [28]. Lactate is correlated with intravascular volume sufficiency and tissue hypoxia. Perioperative blood lactate levels were found to be associated with postoperative complications and the length of hospital stay [29]. In our study, we chose 0.9% NaCl to look at blood lactate levels and to avoid the effect of the ringer lactate solution over the blood lactate level. And lactate levels were found to be statistically significantly higher in the GDFM group at the end of the first hour, although it was found to be similar after surgery. Since the lactate values at this time [1.00 (0.80–1.20) mMol/l] were within the normal reference values, the difference was not considered clinically significant. The lactate levels at 24-h postoperatively were similar in both groups. Therefore, we conclude that lactate levels, which indicate organ perfusion, were affected similarly by both fluid management protocols.

After a major abdominal surgery, 13.4% of patients may develop acute kidney injury (AKI) and long-term and non-renal postoperative complications may develop in patients with AKI [30]. In our study, the number of patients who developed oliguria was similar in the postoperative 24-h follow-ups in both groups. There was no significant difference between the two groups when the preoperative and postoperative creatinine values were compared and no significant change was observed in the postoperative creatinine values according to initial values according to the AKI criteria. We considered that both fluid regimens had similar effects on renal function due to these results.

Fluid management could be done with static, dynamic or invasive, non-invasive parameters. The parameters such as HR, ABP, and urine output may not always provide accurate information in terms of volume status. The patient can be hypervolemic or hypovolemic when HR, ABP, and urine output are normal [17]. Although tachycardia is considered as a classical indicator of hypovolemia, intravascular volume assessment according to HR is deprived of sensitivity and specificity due to the common use of beta-adrenergic receptor blockers in elderly patients [31]. The intermittent measurements of CVP are of limited value unless the CVP is low (< 5 mmHg) and it does not support clinical hypovolemia [32]. The role of CVP monitorization in fluid management is controversial since the threshold values of CVP are uncertain, the measurements are affected by many patient-related factors [33]. In the study by Magder and Bafaqeeh, it was concluded that a CVP value of 10 mmHg could represent euvolemia and the increase in ABP was not a good indicator of cardiac response in fluid management [34]. The dynamic parameters, such as SV, PPV, SPV used for GDFM, were found to be superior to the static parameters in evaluating fluid responsiveness, but the superiority of one of them over the other could not be shown [35–40]. For example, there was no significant difference in the postoperative outcomes between SVI-based GDFM group and the group in which zero balance and postoperative normal weight were aimed [41]. Whereas, in another study, intraoperative crystalloid volume was found to be higher with PVI-based fluid therapy compared to the CVP and MAP targeted group [28]. The results of another study were completely in contradiction to this result [42]. The studies conducted reported that the PVI, a non-invasive method, could be a reliable marker for fluid management in hemodynamically stable patients in cardiac and colorectal surgery [43, 44]. In our study, we only ASA I–II patients, may not be as reliable in cases with higher ASA scores undergoing complex surgery in which more blood and fluid replacement are expected in addition to the possible need for invasive monitoring methods. In this kind of patients, inserting a CVP catheter can be a guiding parameter for the monitoring central oxygen venous saturation (ScvO_2_), which shows the O_2_ demand/supply balance.

In conclusion, the PVI-based GDFM, a non-invasive method for intraoperative fluid management of ASA I–II patients undergoing elective colorectal surgery, may be an alternative to CFM, which is performed with invasive monitorization methods. However, further studies are needed to investigate the efficacy and safety in high-risk patients and complex surgeries, which may require advanced monitorization.
